# Power Outage Preparedness and Concern among Vulnerable New York City Residents

**DOI:** 10.1007/s11524-018-0296-9

**Published:** 2018-07-26

**Authors:** Christine Dominianni, Munerah Ahmed, Sarah Johnson, Micheline Blum, Kazuhiko Ito, Kathryn Lane

**Affiliations:** 1 Division of Environmental Health, New York City Department of Health and Mental Hygiene, New York, NY USA; 2 Baruch College Survey Research, Marxe School of Public and International Affairs, Baruch College, City University of New York, New York, NY USA

**Keywords:** Power outage, Preparedness, Vulnerable populations, Climate change

## Abstract

Power outages can impact health, and certain populations may be more at risk. Personal preparedness may reduce impacts, but information on power outage preparedness and risk perception among vulnerable populations is limited. We examined power outage preparedness and concern among New York City residents, including vulnerable populations defined as older adults (≥ 65 years), and respondents with household members who require assistance with daily activities or depend on electric medical devices. A random sample telephone survey was conducted during November–December 2016. Preparedness was defined as having a three-day supply of drinking water, non-perishable food, and a working flashlight. Among all respondents (*n* = 887), 58% were prepared and 46% expressed concern about health. Respondents with electric-dependent household members (9% of all respondents) tended to have higher preparedness (70 vs. 56% of respondents without electric-dependent household members). Among this group, only 40% reported being registered with a utility company to receive early notification of outages. While the subgroup sample was small, respondents with registered electric-dependent household members had lower preparedness than those with non-registered users (59 vs. 76%). Respondents with household members who needed assistance had comparable levels of preparedness to respondents without someone who needed assistance (59 vs. 57%). Older adults had greater preparedness than younger adults (65 vs. 56%). Health concerns were greater among all vulnerable groups than the general population. Levels of preparedness varied among vulnerable respondents, and awareness of power outage notification programs was low. Our findings highlight the need to increase awareness and preparedness among at-risk people.

## Introduction

Power outages can greatly impact public health. While major outages are relatively infrequent in New York City (NYC), the city has experienced health impacts due to power outages, most notably the August 2003 Northeast outage that affected the entire city, home to over eight million residents [1], and the outages caused by Superstorm Sandy in October 2012 [2]. The 2003 power outage has been associated with increased risk of all-cause mortality [3] and respiratory hospitalizations [4]. It also disrupted refrigeration, potable water pumping equipment in buildings with more than six floors, and elevators—compromising the safety of water and food and stranding residents in their apartments [5, 6]. Superstorm Sandy disrupted power for over two million city residents, many of whom were without power, water, and heat for an extended period of time [2]. Some of the direct health effects of Superstorm Sandy included disruptions in dialysis treatments, increased emergency room visits for renal- and respiratory-related conditions, and increased carbon monoxide exposures and poisonings [7, 8].

NYC power outages can also be confined to smaller areas, such as the July 1999 outage in Northern Manhattan and the July 2006 outage in Western Queens, which roughly impacted 200,000 residents each [9, 10]. Both of these outages occurred with heatwaves, when there is peak electricity demand from air conditioning [11]; however, winter month outages from wind, ice, and snow can also occur. These smaller, localized outages may have health effects comparable to major events but on a smaller scale [12]. As the climate changes, higher average summer temperatures and more heat waves could lead to greater electricity demand and an increase in severe storms that could damage energy infrastructure, resulting in more outages [13]. Without adaptation, future health impacts of power outages may increase.

People at greater risk of power outage health impacts include older adults, people who rely on a caregiver to perform daily activities, and those who depend on electric medical devices [4, 7, 14, 15]. These populations often have more health problems, medication needs, and/or limited mobility [16, 17]. Personal preparedness, especially for those most at risk, may reduce health impacts. Federal agencies recommend developing a preparedness plan [18] and direct the general public to have several items on hand, including a working flashlight with extra batteries, 3-day supply of non-perishable food and drinking water for each household member, and extra medications. Having critical items on hand can help mitigate the effects of power outages and help people to safely shelter in place. A preparedness plan should also include the identification of exit routes from homes and neighborhoods, and a meeting spot for all household members [19]. This can support household readiness for events that may require home evacuation, such as outages associated with severe storms with flooding. For people who depend on electric medical equipment, preparedness also includes signing up with their utility companies to receive notification before a power outage [20] and having a back-up supply of power for their equipment.

While studies have assessed preparedness for general disasters [21–26], few have focused specifically on power outages [27–29], and there is limited knowledge on the usage of utility programs for people on electric medical equipment [30]. In addition, power outage concern may influence personal preparedness. To address these knowledge gaps, we conducted a random sample telephone survey to better understand power outage preparedness and concern among NYC residents, including vulnerable populations. We also aimed to assess awareness of the utility programs among respondents with a household member dependent on electric medical equipment. This information may assist emergency preparedness planning and improve health-risk communications and targeted guidance.

## Methods

The NYC Department of Health and Mental Hygiene (DOHMH), in collaboration with Baruch College Survey Research, conducted a random sample telephone survey among NYC residents 18 years or older between November 18 and December 23, 2016. The NYC DOHMH Institutional Review Board approved this study as exempt research.

The landline telephone sample was based on a random digit dial (RDD) design which draws numbers from all existing landline telephone exchanges in the five boroughs of NYC, giving all listed and unlisted phone numbers a proportionate chance of being included. Respondents in the landline sample were selected randomly within the household based on the most recent birthday. This sample was supplemented by a RDD cell phone sample, based on numbers identified as active cell phones in the five NYC boroughs. Respondents were offered the option of being interviewed in English or Spanish. Participation was voluntary and anonymous. Data were weighted to the United States 2010 Census population data for age, sex, race, Hispanic origin, and borough for NYC adults. The estimated average sample tolerance for data from the poll is ± 3.3 percentage points for the full sample.

The survey consisted of 22 closed-ended questions to collect data on respondent characteristics, household preparedness, and concerns related to power outages, and to identify vulnerable groups. Respondents who reported their households were prepared for a power outage were defined as perceiving their households to be prepared. We defined respondents as prepared if they reported all three preparedness items listed in guidelines for the general public [18], including a working flashlight, 3-day supply of food that would not spoil, and 3-day supply of drinking water. We focused on having emergency preparedness supplies rather than emergency preparedness plans because home evacuations are often not needed during a typical power outage in NYC [19]. Respondents who expressed being very or somewhat concerned that power outages could cause injury or illness to themselves or someone in their household were defined as having a health concern.

We defined three subpopulations as higher risk—older adults (≥ 65 years old); respondents with someone in their households who needed assistance with daily activities, such as eating, bathing or dressing, and would require help leaving the house during a power outage, excluding healthy children; and respondents with someone in their households who was dependent on an electric medical device. Household preparedness and health and safety concerns were further assessed among these vulnerable groups.

Descriptive analysis included unweighted frequencies and calculation of weighted percentages and confidence intervals. *T* tests were used to assess statistically significant differences between groups. All analyses were carried out using SUDAAN 11.0.1 software.

## Results

### Descriptive Characteristics of All Respondents

A total of 887 people responded to the survey. The survey cooperation rate was 49%, the response rate was 12%, and the contact rate was 28% [31]. Approximately half of the surveys were conducted on a landline (47%) and the rest were conducted on cellphones. The majority were conducted in English (91%).

The characteristics of the survey respondents are shown in Table 1. The distribution of respondent demographic characteristics by gender, age, and race and ethnicity were comparable to the 2010 Census data for adult New Yorkers [32]. Multi-family buildings (56%), including walk-ups and those with elevators, were the most common residence type.

**Table 1:** Characteristics of survey respondents

		Unweighted *N*	Weighted %^a^
Gender	Male	360	46
	Female	507	53
	Other	10	1
	Missing/refused	10	
Age	18–29	136	23
	30–49	297	37
	50–64	230	23
	65+	207	17
	Missing/refused	17	
Race/Ethnicity	White, non-Hispanic	332	34
	Black, non-Hispanic	225	21
	Hispanic	161	28
	Asian	71	15
	Something else	40	3
	Missing/refused	58	
Household members	1	173	12
	2+	696	88
	Missing/refused	18	
Children < 12 years	0	629	69
	1+	250	31
	Missing/refused	8	
Household income	< $30,000	161	23
	$30,000 to < $50,000	155	15
	$50,000 to < $100,000	179	28
	≥ $100,000	182	34
	Missing/refused	210	
Residence building type	One or two family home	316	44
	Multi-family building	508	56
	Other/missing/refused	63	

^a^Individual weights were used and missing data were not included in percentages

### Power Outage Preparedness and Concern among All Respondents

Less than half of the respondents perceived their households as prepared for a power outage (46%). However, 89% of all respondents reported having a working flashlight, 71% had a 3-day supply of food that would not spoil, 59% had a 3-day supply of drinking water, and 58% had all three items defined as actual preparedness. When asked how news or information would be received during a power outage, most reported they would use their mobile devices for internet or texts (59%), followed by radios or battery-operated televisions (35%) and paper newspapers or magazines (6%). A little less than half of the respondents were concerned about health (46%) during a power outage.

Actual preparedness was lower among Hispanic respondents (45%, *p* = 0.03, Table 2), those with household income less than $30,000 (45%, *p* = 0.05), and those who live in multi-family buildings (51%, *p* = 0.02). Additionally, Hispanic respondents who completed the survey in Spanish had lower actual preparedness compared to those who completed the survey in English (29 vs. 52%, *p* = 0.03). Perceived preparedness did not differ drastically by any of these characteristics. Respondents who had young children in their household had lower perception of and actual preparedness than those without young children (39 vs. 50%, *p* = 0.03 and 44 vs. 63%, *p* < 0.01, respectively). Black and Hispanic respondents and those with household incomes less than $100,000 expressed greater concern about health during a power outage (all *p* < 0.05).

**Table 2:** Frequency of preparedness and concern by survey respondent characteristics

	Perceived^a^ Prep. weighted % (95% CI)^d^	Actual^b^ Prep. weighted % (95% CI)^d^	Health^c^ Concern weighted % (95% CI)^d^
Total	46 (42, 50)	58 (53, 62)	46 (42, 50)
Race/Ethnicity			
White, Non-Hispanic (ref)	46 (39, 53)	59 (52, 66)	38 (32, 45)
Black, Non-Hispanic	45 (37, 54)	65 (56, 73)	54 (46, 62)^e^
Hispanic	50 (40, 60)	45 (35, 56)^e^	51 (42, 60)^e^
Asian	41 (28, 55)^f^	61 (45, 75)^f^	43 (31, 57)^f^
Household income			
≥ $100,000 (ref)	42 (34, 51)	60 (51, 69)	36 (28, 45)
$50,000 to < $100,000	40 (31, 50)	58 (47, 68)^f^	48 (39, 58)^e^
$30,000 to < $50,000	48 (38, 59)^f^	53 (42, 64)^f^	52 (42, 61)^e^
< $30,000	39 (29, 49)^f^	45 (35, 57)^e,f^	53 (43, 62)^e^
Residence building type			
One or two family home (ref)	46 (39, 54)	63 (55, 70)	49 (42, 56)
Multi-family building	46 (40, 51)	51 (45, 57)^e^	44 (39, 50)
Children < 12 years			
0 (ref)	50 (44, 55)	63 (58, 68)	45 (40, 50)
1+	39 (31, 47)^e^	44 (36, 53)^e^	48 (41, 56)
Lost power in Superstorm Sandy			
No (ref)	47 (41, 52)	57 (51, 62)	46 (41, 51)
Yes	46 (38, 54)	62 (54, 69)	46 (39, 54)

^a^Respondents reporting their households were prepared for a power outage in NYC

^b^Respondents having a working flashlight, 3-day supply of food that would not spoil, and 3-day supply of drinking water

^c^Respondents reporting being very or somewhat concerned that power outages could cause injury or illness

^d^Individual weights were used

^e^Proportion significantly differed when compared to reference group (*p* ≤ 0.05)

^f^Estimate should be interpreted with caution. The 95% confidence interval half-width is > 10 or the sample size is < 50, making the estimate potentially unreliable

The number of respondents who reported losing power during Superstorm Sandy was 29%, consistent with citywide estimates [2]. However, losing power during Superstorm Sandy did not influence perceived or actual preparedness and health concerns.

### Preparedness and Concern among At-Risk Respondents

Among all respondents, a quarter had a household member who needed assistance with daily activities (25%), 17% were older adults, and 9% said there was someone in their household who depended on an electric medical device (Table 3). All three at-risk populations overlapped with 38% of all respondents belonging to at least one at-risk group. There was significant overlap between respondents with household members needing assistance and respondents with electric-dependent household members (for example, 61% of respondents with electric-dependent household members also had a household member who needed assistance).

**Table 3:** Overlap among different at-risk populations

	Total	Older adults	Household member needing assistance	Household member is electric-dependent
	Unweighted N (weighted %^a^)	Weighted % (95% CI)^a^	Weighted % (95% CI)^a^	Weighted % (95% CI)^a^
Older adults				
65+	207 (17)	NA	19 (14, 25)	22 (13, 34)^c^
< 65	663 (83)	NA	16 (13, 19)	16 (14, 19)
Household member needing assistance				
Yes	222 (25)	29 (22, 37)	NA	61 (47, 74)^b, c^
No	656 (75)	25 (21, 29)	NA	22 (19, 26)
Household member is electric-dependent				
Yes	77 (9)	12 (7, 18)^c^	21 (15, 30)^b, c^	NA
No	807 (91)	8 (6, 12)	5 (3, 7)	NA

^a^Individual weights were used

^b^Proportion significantly differed when compared to non-vulnerable groups (*p* ≤ 0.05)

^c^Estimate should be interpreted with caution. The sample size is < 50 making the estimate potentially unreliable

Older respondents were more likely to live alone (37 vs. 7%, *p* < 0.01, Table 4) and live in a multi-family building (64 vs. 54%, *p* = 0.03) than younger respondents. They were more prepared (65 vs. 56%, *p* = 0.08) and more reported that they would get information from a radio or battery-operated television (52 vs. 31%, *p* < 0.01). Older adults who live alone (*n* = 91) had a lower perception of preparedness compared to older adults not living alone (42 vs. 57%, p = 0.08). All older respondents expressed greater health concern (54 vs. 45%, *p* = 0.05) but concern appeared to be greater among older respondents who live alone (61 vs. 49% of older adults who do not live alone, *p* = 0.13).

**Table 4:** Characteristics, preparedness, and concern of older adults

	Weighted % (95% CI)^a^		Weighted % (95% CI)^a^	
	65+ years (*n* = 207)	< 65 years (ref, *n* = 663)	65+ years living alone (n = 91)	65+ years not living alone (ref, *n* = 112)
Race/Ethnicity				
White, Non-Hispanic	55 (47, 64)^e^	30 (26, 34)	53 (42, 65)^f^	55 (44, 66)
Black, Non-Hispanic	20 (15, 27)^f^	21 (18, 25)	22 (15, 32)^f^	20 (13, 29)
Hispanic	16 (10, 24)^e,f^	30 (25, 35)	19 (11, 32)^f^	14 (7, 26)
Asian	6 (3, 13)^e,f^	17 (13, 21)	3 (1, 13)^f^	8 (3, 19)
Living alone	37 (30, 45)^e^	7 (6, 9)	NA	NA
Children < 12 years	12 (7, 19)^e,f^	35 (31, 40)	NA	NA
Household income < $50,000	39 (31, 48)	38 (33, 43)	NA	NA
Multi-family building	64 (56, 72)^e^	54 (49, 59)	78 (68, 86)^e,f^	58 (46, 68)
Perceived preparedness^b^	51 (42, 59)	45 (40, 50)	42 (31, 54)^f^	57 (44, 68)
Actual preparedness^c^	65 (56, 73)	56 (50, 61)	59 (46, 70)^f^	68 (56, 78)
Getting Information				
Internet/website on mobile device, texts	40 (32, 48)^e^	63 (58, 67)	47 (36, 59)^f^	35 (25, 47)
Radio or battery operated television	52 (43, 60)^e^	31 (27, 36)	45 (34, 57)^f^	56 (44, 67)
Has health concern^d^	54 (46, 62)^e^	45 (40, 49)	61 (50, 71)^f^	49 (38, 60)

^a^Individual weights were used

^b^Respondents reporting their households were prepared for a power outage in NYC

^c^Respondents having a working flashlight, 3-day supply of food that would not spoil, and 3-day supply of drinking water

^d^Respondents reporting being very or somewhat concerned that power outages could cause injury or illness

^e^Proportion of characteristics significantly differed when compared to non-vulnerable groups (*p* ≤ 0.05)

^f^Estimate should be interpreted with caution. Estimate’s relative standard error (a measure of estimate precision) is greater than 30%, the 95% confidence interval half-width is > 10, or the sample size is < 50, making the estimate potentially unreliable

Respondents who reported having someone in the household who needed assistance with daily activities were more likely to be Hispanic (36 vs. 25%, *p* = 0.03, Table 5), have young children in their household (43 vs. 28%, *p* < 0.01), have a lower household income (57 vs. 32%, p < 0.01), and live in a multi-family building (67 vs. 52%, *p* < 0.01) than respondents who did not have someone in their household who required assistance. There was no significant difference in perceived or actual preparedness; however, those with someone in their household who needed assistance expressed greater health concerns related to power outages (65 vs. 39%, p < 0.01).

**Table 5:** Characteristics, preparedness, and concern of respondents with household members needing assistance

	Weighted % (95% CI)^a^	
	Yes (*n* = 222)	No (ref, *n* = 656)
Race/Ethnicity		
White, Non-Hispanic^e^	18 (13, 24)	39 (35, 44)
Black, Non-Hispanic	26 (20, 34)	20 (16, 23)
Hispanic^e^	36 (28, 45)	25 (21, 30)
Asian^f^	16 (11, 24)	14 (10, 19)
Living alone^f^	9 (6, 14)	13 (11, 16)
Children < 12 years^e^	43 (35, 51)	28 (24, 32)
Household income < $50,000^e^	57 (47, 66)	32 (27, 37)
Multi-family building^e^	67 (58, 74)	52 (47, 56)
Perceived preparedness^b^	44 (36, 53)	46 (41, 52)
Actual preparedness^c^	59 (49, 68)	57 (52, 62)
Getting Information		
Internet/website on mobile device, texts	63 (55, 71)	57 (52, 62)
Radio or battery operated television	31 (24, 40)	37 (32, 42)
Has health concern^d,e^	65 (57, 73)	39 (35, 44)

^a^Individual weights were used

^b^Respondents reporting their households were prepared for a power outage in NYC

^c^Respondents having a working flashlight, 3-day supply of food that would not spoil, and 3-day supply of drinking water

^d^Respondents reporting being very or somewhat concerned that power outages could cause injury or illness

^e^Proportion of characteristics significantly differed when compared to non-vulnerable groups (*p* ≤ 0.05)

^f^Estimate should be interpreted with caution. The sample size is < 50 making the estimate potentially unreliable

Respondents who reported having a household member dependent on electrically powered medical equipment were more likely to have a lower household income (57 vs. 36%, *p* = 0.01, Table 6) and live in a multi-family building (70 vs. 54%, *p* = 0.03) compared to those who did not. These respondents were less likely to perceive their households as prepared (32 vs. 47%, p = 0.03) but tended to be actually prepared (70 vs. 56%, *p* = 0.11). Compared to the rest of the sample, they would be more likely to use mobile devices to access information during power outages (70 vs. 58%, *p* = 0.07). They were also more likely to express greater concern about health (74 vs. 44%, *p* < 0.01) during a power outage. Among respondents who reported having someone in their household dependent on electrically powered medical equipment, only 40% (*n* = 25) said that person was registered with a utility company to receive notification before a power outage. These respondents were less likely to have lost power during Superstorm Sandy (18 vs. 39%, *p* = 0.09, Table 7) and had lower actual preparedness (59 vs. 76%, *p* = 0.34). They also expressed greater concern about health (90 vs. 68%, *p* = 0.04) during power outages.

**Table 6:** Characteristics, preparedness, and concern of respondents with household members needing electric medical equipment

	weighted % (95% CI)^a^	
	Yes (*n* = 77)	No (ref, *n* = 807)
Race/ethnicity		
White, Non-Hispanic^f^	31 (20, 45)	34 (30, 38)
Black, Non-Hispanic^f^	17 (9, 30)	22 (19, 26)
Hispanic^f^	35 (22, 51)	27 (23, 31)
Asian^f^	13 (5, 30)	14 (11, 18)
Living alone^f^	11 (5, 20)	13 (11, 15)
Children < 12 years^f^	23 (13, 37)	32 (28, 37)
Household income < $50,000^e,f^	57 (41, 72)	36 (32, 41)
Multi-family building^e,f^	70 (55, 81)	54 (50, 59)
Perceived preparedness^b,e,f^	32 (20, 46)	47 (42, 52)
Actual preparedness^c,f^	70 (52, 83)	56 (51, 61)
Getting information		
Internet/website on mobile device, texts^f^	70 (56, 81)	58 (53, 62)
Radio or battery operated television^f^	30 (19, 44)	35 (31, 40)
Has health concern^d,e,f^	74 (60, 85)	44 (39, 48)

^a^Individual weights were used

^b^Respondents reporting their households were prepared for a power outage in NYC

^c^Respondents having a working flashlight, 3-day supply of food that would not spoil, and 3-day supply of drinking water

^d^Respondents reporting being very or somewhat concerned that power outages could cause injury or illness

^e^Proportion of characteristics significantly differed when compared to non-vulnerable groups (*p* ≤ 0.05)

^f^Estimate should be interpreted with caution. Estimate’s relative standard error (a measure of estimate precision) is greater than 30%, the 95% confidence interval half-width is > 10, or the sample size is < 50, making the estimate potentially unreliable

**Table 7:** Characteristics, preparedness, and concern between respondents with registered electric-dependent household members and respondents with non-registered electric-dependent household members

	Weighted % (95% CI)^a^	
	Registered (*n* = 25)	Non-registered (ref, *n* = 45)
Race/ethnicity		
White, Non-Hispanic^f^	33 (15, 58)	32 (18, 50)
Other^f^	67 (42, 85)	68 (50, 82)
Household income < $50,000^f^	63 (35, 84)	51 (32, 71)
Multi-family building^f^	75 (49, 90)	66 (47, 81)
Lost power in Superstorm Sandy^f^	18 (8, 37)	39 (22, 58)
Perceived preparedness^b,f^	34 (16, 59)	30 (16, 50)
Actual preparedness^c,f^	59 (27, 85)	76 (57, 89)
Getting Information		
Internet/website on mobile device, texts^f^	75 (52, 89)	65 (45, 81)
Radio or battery operated television^f^	25 (11, 48)	35 (19, 55)
Has health concern^d,e,f^	90 (73, 97)^h^	68 (48, 83)

^a^Individual weights were used

^b^Respondents reporting their households were prepared for a power outage in NYC

^c^Respondents having a working flashlight, 3-day supply of food that would not spoil, and 3-day supply of drinking water

^d^Respondents reporting being very or somewhat concerned that power outages could cause injury or illness

^e^Proportion of characteristics significantly differed when compared to non-registered respondents (*p* ≤ 0.05)

^f^All estimates should be interpreted with caution. Estimate’s relative standard error (a measure of estimate precision) is greater than 30% or the sample size is too small (*n* < 50), making the estimate potentially unreliable

## Discussion

Having key items, such as a working flashlight and 3-day supply of water and food, is one important component of a preparedness plan and can make it easier to cope with power outages [18]. However, we found that many of the respondents did not have all of these basic necessities, which is consistent with a national-based survey of emergency preparedness [24]. A survey among residents in Ontario who lost power during the August 2003 power outage found the majority of those who had not assembled an emergency preparedness kit did not think it was important [33]. In NYC, where major power outages are relatively infrequent, power outages may not be perceived as threats to most residents. For at-risk people, a power outage could present more challenges [34] so that they may feel a greater threat to their health or safety; however, having concerns about power outages does not necessarily translate into greater preparedness, suggesting that there are barriers to obtaining and maintaining basic preparedness supplies.

Respondents with electrically dependent household members were identified as more likely to have all three basic preparedness items. A loss of power poses a serious risk of disruption of medical equipment operation, placing those who depend on them in potentially life-threatening situations [34], perhaps driving preparedness levels among this group. However, the perception of preparedness was significantly lower in this group indicating that they viewed preparedness as much more than our definition captured. Preparedness has been shown to be low in this vulnerable population as it includes more challenging aspects such as ensuring the medical equipment runs during the outage [29].

Utility power outage notification programs can sometimes provide electric medical equipment users advanced warning; however, our findings indicated awareness of these programs was low as has been previously observed [30]. Those who registered for the program also had greater health concerns. However, we also observed a non-significant trend in lower preparedness among these respondents, possibly because they may view being on the registry as sufficient preparedness, or because they do not expect to shelter in place during an extended outage. While we can only speculate on the reasons for lower preparedness in this specific group, these findings highlight the importance of encouraging preparedness among all households with electric medical equipment users, including having and maintaining back-up sources of power, regardless of whether they have signed up with their utility company.

Preparedness among respondents who had someone in their household who needed assistance with daily activities was comparable to those who did not, consistent with studies in Southeastern Pennsylvania [35] and North Carolina [36]. Caregivers may face additional challenges in preparedness due to the medical needs of family members who require assistance [25, 37]. There may also be other competing priorities, such as care for young children, which was prevalent in this group. In addition, our findings pointed toward socioeconomic barriers among this at-risk group. They were more likely to have lower household income which may limit ability to purchase supplies. They were also more likely to be Hispanic, possibly reflecting limited availability of or access to preparedness resources, including materials in Spanish, or cultural differences that influence perception by respondents and communication of risk by emergency response and preparedness planners [38–41]. Addressing these limitations may provide one way to increase preparedness among this vulnerable group in NYC.

Our findings pointed toward lower perception of preparedness among the most vulnerable older adults, those who live alone. Besides being at greater risk of social isolation [42], their greater health concerns suggest they may have more health problems, factors that can impede their ability to prepare for outages [24, 43]. In addition, they may have more immediate concerns than preparing for an outage, suffer from anxiety when thinking of emergencies, or feel that such emergencies are out of their control [23]. Lower preparedness could have significant implications for older adults in general who are at greater risk of falls [44], heat illness and death [45], and other adverse health effects that could be exacerbated by power outages [15]. Encouraging older adults to stock basic preparedness supplies may help minimize the risk of some of these outcomes, such as using a flashlight to prevent fall-related injuries and staying hydrated, especially during hot weather outages.

We found that the vulnerable groups in our study were more likely to live in a multi-family building which may present additional challenges for them during power outages, particularly if they live on higher floors. They may lose access to safe drinking water and be harder to reach due to impaired water pumping equipment and elevators [6]. Community-based programs that encourage people to check on at-risk neighbors are additional ways to promote power outage safety among residents emergency responders cannot easily reach.

Mobile devices for accessing the internet or receiving texts were generally the most common way information would be obtained during a power outage. However, a loss of power could make it difficult to charge devices and disrupt services, rendering them useless. As a previous study showed, traditional sources of information, such as TV and radio, were more heavily relied on among New Jersey respondents impacted by Superstorm Sandy [46]. Other means of providing information are needed and should be clearly stated in the messages that go out prior to an outage, if possible, so people will be able to access information during the outage. Prior to an outage, messaging developed for use on mobile devices must be clear, available in multiple languages, and easy to find, especially if sent through links in texts.

Respondents who lost power during Superstorm Sandy were not more likely to perceive their households as prepared or actually be prepared. Findings on the relationship between prior disaster experience and preparedness have been mixed [47–49]. A wide variety of factors likely influence this relationship, including the frequency and severity of outages experienced. Some respondents who lost power during Sandy may have lost it for only a short period of time, while some may have lost power for weeks. Respondents may have not have suffered any injuries or illness related to the power outage, which may reduce inclinations to prepare for future events. In addition, there may be misclassification of prior outage experience because we only asked about Sandy. Respondents may have had a range of previous power outage experience, in addition to other disaster experiences, such as the September 2001 World Trade Center terrorism attack, which could have influenced preparedness [47].

Our study had several important limitations. While our overall sample was large, some of our subgroup samples were small, likely resulting in unreliable estimates. Since most NYC outages do not warrant evacuations, we focused our questions on having supplies as an indicator of basic preparedness. However, our questions may have only captured these supplies as part of normal household goods and not necessarily with the intent of preparedness, in which case timing of when the goods were obtained in relation to when the survey was conducted could impact how preparedness was captured [50]. We also did not ask about certain health-relevant topics including keeping a supply of back-up batteries for medical equipment, access to emergency refills for prescription medications, or knowledge of home food safety during outages. Our survey did not inquire about the respondents’ health status or if they were the household member needing assistance or on an electrically powered medical device. As a result, we could not directly assess if health status was associated with levels of concern and preparedness. We also asked household-level questions for certain topics, limiting our ability to draw individual conclusions.

## Conclusions

A power outage in NYC could have health impacts, especially for the most at-risk residents. While concern about power outages was higher among vulnerable groups, preparedness was still low for some, highlighting a need for targeted education and resources prior to an outage. Our findings also pointed to low awareness of power outage notification programs available through utility companies. As such, additional work can be done to encourage preparedness, including raising awareness about how to register for utility company power outage notification programs. Our study established an understanding of basic power outage preparedness among the general population and some vulnerable groups in NYC. More detailed surveys or focus groups among these vulnerable populations may help further understand what drives preparedness and identify ways to improve emergency preparedness planning in protecting populations most at-risk to illness, injury, or death associated with power outages.
